# Modeling and Closed Loop Flight Testing of a Fixed Wing Micro Air Vehicle

**DOI:** 10.3390/mi9030111

**Published:** 2018-03-04

**Authors:** Harikumar Kandath, Jinraj V. Pushpangathan, Titas Bera, Sidhant Dhall, M. Seetharama Bhat

**Affiliations:** 1 School of Electrical and Electronic Engineering, Nanyang Technological University, Singapore 639798, Singapore; btitas@ntu.edu.sg; 2 Department of Aerospace Engineering, Indian Institute of Science, Bengaluru 560012, India; jinrajaero@gmail.com (J.V.P.); sidhant.dhall@gmail.com (S.D.); msbdcl@aero.iisc.ernet.in (M.S.B.)

**Keywords:** flight testing, lateral dynamics, longitudinal dynamics, micro air vehicle, propeller effects, wind tunnel testing

## Abstract

This paper presents the nonlinear six degrees of freedom dynamic modeling of a fixed wing micro air vehicle. The static derivatives of the micro air vehicle are obtained through the wind tunnel testing. The propeller effects on the lift, drag, pitching moment and side force are quantified through wind tunnel testing. The dynamic derivatives are obtained through empirical relations available in the literature. The trim conditions are computed for a straight and constant altitude flight condition. The linearized longitudinal and lateral state space models are obtained about trim conditions. The variations in short period mode, phugoid mode, Dutch roll mode, roll subsidence mode and spiral mode with respect to different trim operating conditions is presented. A stabilizing static output feedback controller is designed using the obtained model. Successful closed loop flight trials are conducted with the static output feedback controller.

## 1. Introduction

The micro air vehicles (MAV) are small aircraft with a wingspan of 150 mm and a maximum takeoff weight less than 200 g [1,2]. MAV can be deployed in confined air spaces where it is arduous for bigger UAV (unmanned air vehicle) to operate. The three categories of MAVs are the fixed wing, flapping wing and rotary wing [3,4,5]. Among the three types of MAVs, fixed wing MAVs have higher flight velocity and longer endurance when compared to flapping wing and rotary wing counterparts. Fixed wing MAVs face many unique challenges that make their design and development difficult. The development of a fully autonomous fixed wing MAV is a very challenging task. The fixed wing MAVs operate in low Reynolds number (Re) regime where many complex flow phenomena take place within the boundary layer. The Separation, transition, and reattachment of air flow can all occur within a short distance along the chord line of the wing [1].

Since MAVs are lightweight, low moment of inertia air vehicles, they are very much susceptible to atmospheric disturbances [6]. A robust feedback control is required for MAVs to operate in such adverse atmospheric conditions. A feedback controller is designed based on the mathematical model describing the dynamics of the MAV. To construct the mathematical model of the MAV, the forces and moments acting on MAV are estimated through the wind tunnel testing and computational fluid dynamic (CFD) analysis. MAVs have low aspect ratio wing (AR<3). The forces generated by propeller wake is significant for MAVs [7]. Hence, a conventional aircraft modeling techniques that do not take into account of propeller wake are inadequate to model the aerodynamic forces acting on MAV. The aerodynamic effects of propeller flow on MAV and small UAV are studied in [7,8,9,10,11,12,13,14]. The influence of propeller wake on the lift, drag and wing stall characteristics is reported in [7,8,9,10,11,12,13,14]. There is an increase in lift force generated by rotating propeller when compared to stationary propeller conditions. The drag force with a rotating propeller is considerably higher than that in the case of the wing without the rotating propeller. The stall of the wing is delayed by 10${}^{\circ }$ of an angle of attack (α) with the aerodynamic effects of rotating propeller. In [15], a six degree of freedom nonlinear model for MAV is developed without including the propeller effects. Thereafter, a linear model which includes terms which are often neglected in the linear model of a bigger aircraft is constructed by linearizing the six degrees of freedom nonlinear model around a trim point. A specific nonlinear model of a gun launched MAV (GLMAV) for hover and near-hover flight conditions are presented in [16]. In [17], the aerodynamic modeling of the longitudinal aerodynamics of MAV at the high angle of attack under unsteady conditions is carried out. For a 75 mm wingspan fixed wing nano air vehicle (NAV), in [18], the longitudinal, lateral and coupled models excluding propeller effects computed using CFD tools are presented.

This paper explains in detail the nonlinear six degrees of freedom (6DOF) dynamic modeling of a fixed wing MAV. A very brief explanation of the dynamics of the MAV considered in this paper is presented in [19]. The lift, drag, pitching moment and side force is computed from wind tunnel testing with rotating propeller. The effect of propeller wake in the lift, drag, pitching moment and side force is quantified with explicit equations obtained through curve fitting the wind tunnel test data. The dynamic thrust of motor-propeller is estimated by measuring the axial force with rotating propeller and stationary propeller. Some of the static derivatives and the dynamic derivatives are obtained using empirical relations mentioned in [20]. The trim conditions for straight and constant altitude flight are presented for the entire flight envelope. The linear longitudinal and lateral state space model and corresponding transfer function model are presented. A static output feedback controller is designed for the obtained model and closed loop flight testing is conducted in the presence of wind disturbances. The flight test data shows satisfactory stability characteristics of the MAV flight.

The paper is organized as follows. The nonlinear dynamic model of the MAV is presented in Section 2. Wind tunnel test results are discussed in detail in Section 2. The flight envelope of MAV and trim conditions for straight and constant altitude flight is given in Section 3. Linear longitudinal and lateral state space model of the MAV is presented in Section 4. Section 5 gives controller design details and closed loop flight test results followed by conclusions in Section 6.

## 2. Nonlinear Dynamic Model of MAV

**Figure 1:**
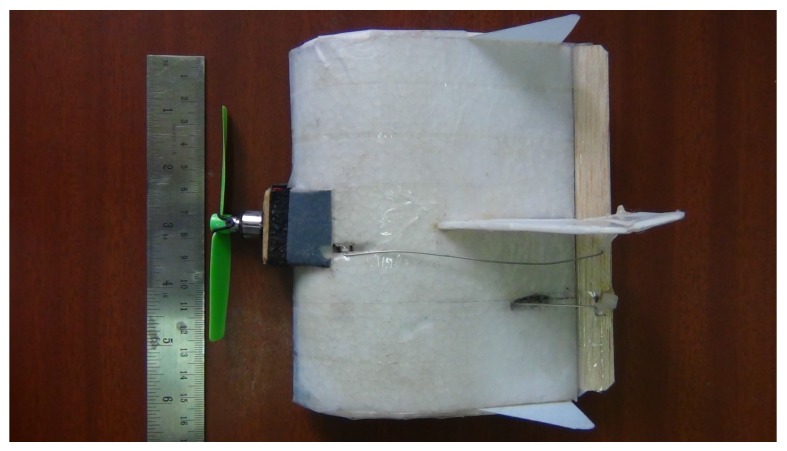
150 mm wingspan MAV.

This section develops the nonlinear dynamic model of 150 mm MAV, named KH2013A shown in Figure 1 with specifications given in Table 1 [19]. The details of the force and moment coefficients used in this section are given in Appendix A.

**Figure 2:**
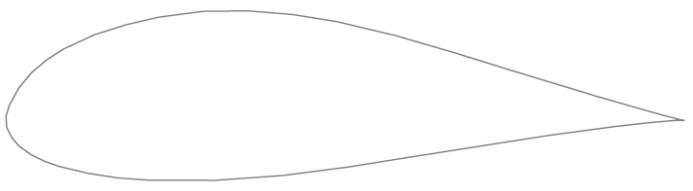
Eppler-387 airfoil.

The MAV is a flying wing aircraft with autopilot hardware and servo motors for actuating control surfaces placed inside the wing. The airfoil used is modified version of Eppler-387 (E387), suitable for low Reynolds number flyers. The thickness of the conventional E387 airfoil of about 9% is increased to 25% to accommodate the autopilot hardware and other components. The profile of the airfoil used in MAV is shown in Figure 2. The specifications of the airfoil are given in Table 2.

The force equations, moment equations and kinematic equations for position and orientation constitutes the six degree of freedom nonlinear model of the MAV. The equations described in this section is explained in more detail in [21]. Let $\bar{V}={\left[u,\;\;v,\;\;w\right]}^{T}$ denote the velocity components of the MAV along the body axis and $\bar{P}={\left[x,\;\;y,\;\;z\right]}^{T}$ be the position of MAV in inertial axis. Let $\omega ={\left[p,\;\;q,\;\;r\right]}^{T}$ denote the angular velocity along the body axis and $\bar{E}={\left[\phi ,\;\;\theta ,\;\;\psi \right]}^{T}$ be the Euler angles. The vector form of force equation is given in (1).

(1) $$\dot{\bar{V}}=\omega \times \bar{V}+\frac{F_{g}+F_{a}+F_{t}}{m}$$

where $F_{g}$ is the gravitational force, $F_{a}$ is the aerodynamic force and $F_{t}$ is the propulsive force. The gravitational force is given in (2).

(2) $$F_{g}={\left[-mgsin\theta ,\;\;mgcos\theta sin\phi ,\;\;mgcos\theta cos\phi \right]}^{T}$$

where *m* is the mass of MAV and *g* is the acceleration due to gravity. Lift and drag forces contribute to the aerodynamic forces acting on MAV and is given in (3).

(3) $$F_{a}={\left[X_{a},\;\;Y_{a},\;\;Z_{a}\right]}^{T}$$

where $X_{a}$, $Y_{a}$ and $Z_{a}$ denotes the aerodynamic forces along body *X* axis, *Y* axis and *Z* axis of the MAV respectively. The expression for $X_{a}$ is given in (4).

(4) $$X_{a}=\left(0.5\rho {V_{a}}^{2}S\right)(C_{L}sin\alpha -C_{D}cos\alpha +\left(C_{Lq}sin\alpha -C_{Dq}cos\alpha \right)\left(\frac{0.5\bar{c}}{V_{a}}\right)q$$

where ρ is the density of air, $V_{a}$ is the MAV velocity magnitude, *S* is the wing area and $\bar{c}$ is the chord length. Similarly the expression for $Y_{a}$ and $Z_{a}$ are given in (5) and (6) respectively.

(5) $$\begin{array}{ccc} Y_{a} & = & \left(0.5\rho {V_{a}}^{2}S\right)\left(C_{y}+C_{yp}\left(\frac{0.5b}{V_{a}}\right)p+C_{yr}\left(\frac{0.5b}{V_{a}}\right)r\right) \end{array}$$

(6) $$Z_{a}=\left(0.5\rho {V_{a}}^{2}S\right)(-C_{L}cos\alpha -C_{D}sin\alpha +\left(-C_{Lq}cos\alpha -C_{Dq}sin\alpha \right)\left(\frac{0.5\bar{c}}{V_{a}}\right)q$$

The propulsive force acts only along body *X* axis as the thrust line of the propeller is aligned with body *X* axis. Hence the propulsive force of the MAV is written as given in (7). In this, T denotes the dynamic thrust and is estimated from wind tunnel tests.

(7) $$F_{t}={\left[T,\;\;0,\;\;0\right]}^{T}$$

The moment balance equations along body *X* axis, *Y* axis and *Z* axis of the MAV is given in (8)–(10) respectively.

(8) $$\begin{array}{ccc} \dot{p} & = & t_{1}pq-t_{2}qr+t_{3}L+t_{4}N \end{array}$$

(9) $$\begin{array}{ccc} \dot{q} & = & t_{5}pr-t_{6}\left(p^{2}-r^{2}\right)+\frac{M}{J_{yy}} \end{array}$$

(10) $$\begin{array}{ccc} \dot{r} & = & t_{7}pq-t_{1}qr+t_{4}L+t_{8}N \end{array}$$

where *L*, *M* and *N* are rolling moment, pitching moment and yawing moment respectively and is given in (11)–(13) respectively.

(11) $$L=0.5\rho {V_{a}}^{2}Sb\left(C_{l\beta }\beta +C_{lp}\left(\frac{0.5b}{V_{a}}\right)p+C_{lr}\left(\frac{0.5b}{V_{a}}\right)r+C_{l\delta r}\delta_{r}\right)-\tau_{c}$$

(12) $$M=0.5\rho {V_{a}}^{2}S\bar{c}\left(C_{m}+C_{mq}\left(\frac{0.5c}{V_{a}}\right)q\right)$$

(13) $$N=0.5\rho {V_{a}}^{2}Sb\left(C_{n\beta }\beta +C_{np}\left(\frac{0.5b}{V_{a}}\right)p+C_{nr}\left(\frac{0.5b}{V_{a}}\right)r+C_{n\delta r}\delta_{r}\right)$$

The parameters $t_{1}$ to $t_{8}$ mentioned in (8)–(10) are constants depending on the moment of inertia of the MAV. The parameters $t_{1}$ to $t_{8}$ are combinedly represented by a vector $t_{m}$ as given in (14). The expression for $t_{m}$ is given in (15).

(14) $$t_{m}=\left[t_{1},\;\;t_{2},\;\;t_{3},\;\;t_{4},\;\;t_{5},\;\;t_{6},\;\;t_{7},\;\;t_{8}\right]$$

(15) $$t_{m}=\left[\frac{J_{xz}\left(J_{xx}-J_{yy}+J_{zz}\right)}{J_{m}},\;\frac{J_{zz}\left(J_{zz}-J_{yy}\right)+J_{xz}J_{xz})}{J_{m}},\;\frac{J_{zz}}{J_{m}},\;\frac{J_{xz}}{J_{m}},\;\frac{J_{zz}-J_{xx}}{J_{yy}},\;\frac{J_{xz}}{J_{yy}},\;\frac{J_{xx}\left(J_{xx}-J_{yy}\right)+J_{xz}J_{xz}}{J_{m}},\;\frac{J_{xx}}{J_{m}}\right]$$

where $J_{xx}$, $J_{yy}$ and $J_{zz}$ are moment of inertia along body *X* axis, *Y* axis and *Z* axis of the MAV. The term $J_{xz}$ is the product of inertia and $J_{m}$ is given in (16).

(16) $$J_{m}=J_{xx}J_{zz}-J_{xz}J_{xz}$$

The relation between body angular rates (ω) and Euler angles ($\bar{E}$) is given below

(17) $$\dot{\bar{E}}=R_{1}\left(\bar{E}\right)\omega$$

where the rotation matrix $R_{1}\left(\bar{E}\right)$ is given in (18).

(18) $$R_{1}\left(\bar{E}\right)=\left(\begin{array}{ccc} 1 & sin\phi tan\theta & cos\phi sec\theta \\ 0 & cos\phi & -sin\phi \\ 0 & sin\phi sec\theta & cos\phi sec\theta \end{array}\right)$$

The velocity component along the body axis is multiplied by the rotation matrix to yield velocity components along the inertial axis. The relation is given in (19).

(19) $$\dot{\bar{P}}=R_{2}\left(\bar{E}\right)\bar{V}$$

where the rotation matrix $R_{2}\left(\bar{E}\right)$ is given in (20).

(20) $$R_{2}\left(\bar{E}\right)=\left(\begin{array}{ccc} cos\theta cos\psi & sin\phi sin\theta cos\psi -cos\phi sin\psi & cos\phi sin\theta cos\psi +sin\phi sin\psi \\ cos\theta sin\psi & sin\phi sin\theta sin\psi +cos\phi cos\psi & cos\phi sin\theta sin\psi -sin\phi cos\psi \\ -sin\theta & sin\phi cos\theta & cos\phi cos\theta \end{array}\right)$$

To summarize, (1), (8), (9), (10), (17) and (19) constitutes the 6DOF nonlinear dynamic equations of MAV.

### 2.1. Estimation of Static Derivatives and Control Derivatives from Wind Tunnel Tests

The wind tunnel tests for the MAV was conducted at National Aerospace Laboratories (NAL). The specifications of the open circuit wind tunnel used for testing is given in Table 3.

**Figure 3:**
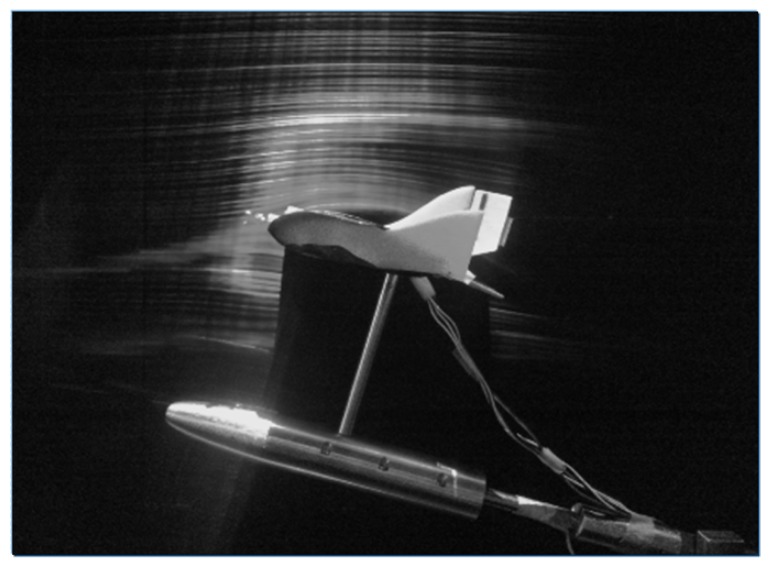
MAV undergoing wind tunnel tests.

The MAV undergoing wind tunnel testing is shown in Figure 3.

The lift force (*L*), side force (*Y*) and aerodynamic pitching moment (*M*) are measured in wind tunnel with rotating propeller. The propeller used here is GWS 5×3 (5-inch diameter and 3-inch pitch) along with Hobbyking AP05 BLDC motor weighing 5.4 g. The specifications of the propeller is given in Table 4 [22]. The static thrust of the propeller with AP05 BLDC motor at an input voltage of 8.4 V is measured using a load cell and is given in Table 5.

**Figure 4:**
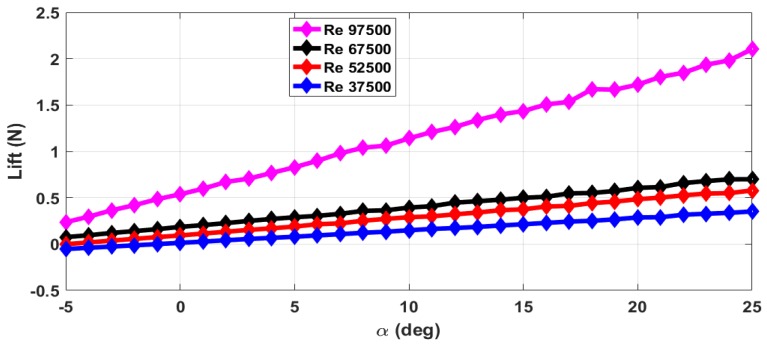
Lift force for zero elevator deflection and zero propeller RPM.

**Figure 5:**
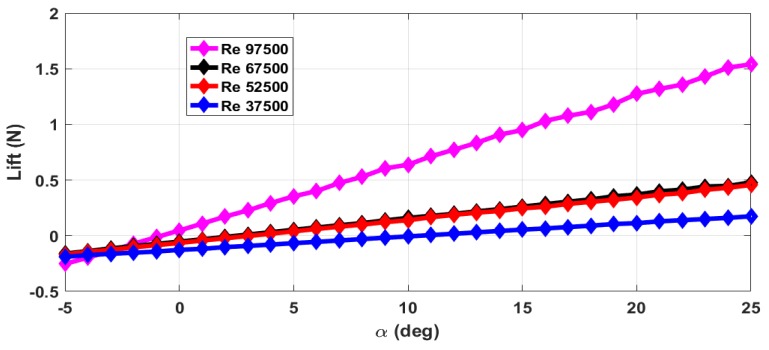
Lift force for −25${}^{\circ }$ elevator deflection and zero propeller RPM.

**Figure 6:**
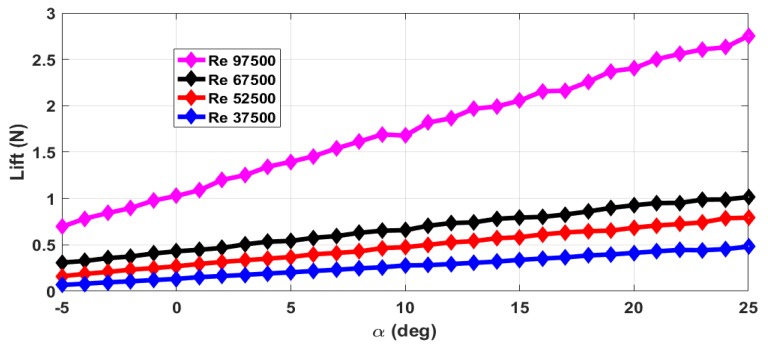
Lift force for +15${}^{\circ }$ elevator deflection and zero propeller RPM.

**Figure 7:**
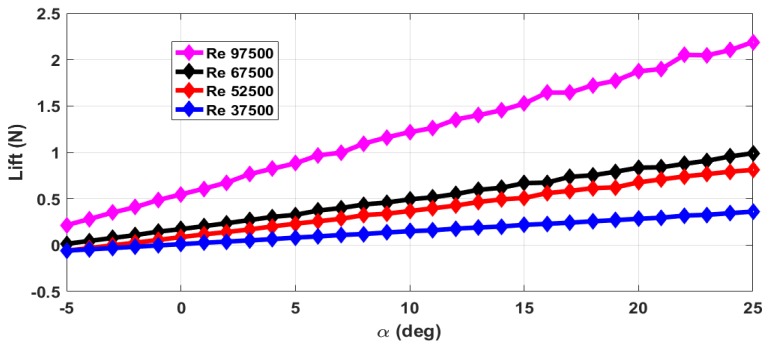
Lift force for zero elevator deflection and a propeller RPM of 8000.

**Figure 8:**
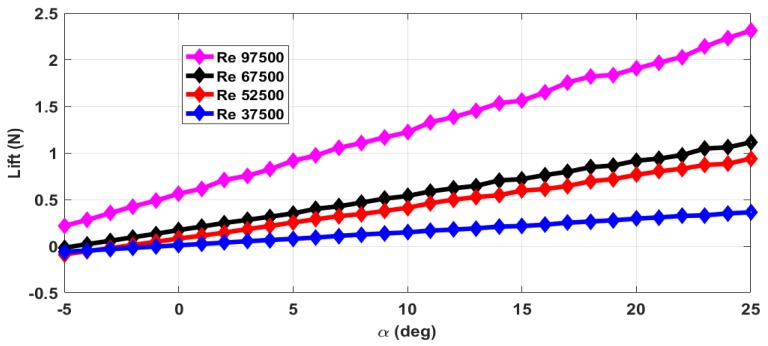
Lift force for zero elevator deflection and a propeller RPM of 12,000.

The thrust force (*T*) and drag force (*D*) are estimated from the axial forces measured with rotating propeller and with the stationary propeller. The maximum RPM (rotations per minute) value is 12,000 with airflow. The stall angle of attack ($\alpha_{stall}$) is not captured in the wind tunnel tests as the there were provisions for varying the angle of attack from −5${}^{\circ }$ to +25${}^{\circ }$ only. The air velocity is varied from 5 m/s to 13 m/s and measurements are taken. This corresponds to the Reynolds number range of 37,500 to 97,500. The lift force obtained with zero elevator deflection and zero propeller RPM is shown in Figure 4. The measurements are taken for an interval of 1${}^{\circ }$ of α and are marked in all the plots. From Figure 4, we can see that the total lift force generated increases with increase in Reynolds number. The plot of lift generated when the elevator is deflected 25${}^{\circ }$ upwards is shown in Figure 5. From Figure 4 and Figure 5, we can see that there is a reduction in the lift force when the elevator is deflected upwards as it reduces the effective camber of the airfoil. Figure 6 shows the lift generated for 15${}^{\circ }$ downward elevator deflection. From Figure 4 and Figure 6, we can see that there is an increase in the lift force when the elevator is deflected downwards as it increases the effective camber of the airfoil. The amount of lift force generated is significantly influenced by the propeller wake as the ratio of the propeller diameter to the wingspan is very high (0.68) when compared to the other UAVs. The plot of the lift force generated for a propeller RPM of 8000 and 12,000 is given in Figure 7 and Figure 8 respectively. While comparing Figure 4 and Figure 8, we can see that there is at least 10% increase in the lift force generated under the influence of propeller wake.

The relation between coefficient of lift ($C_{L}$) and total lift force is given in Equation (21).

(21) $$L=C_{L}\left(0.5\rho V_{a}^{2}S\right)$$

The coefficient of lift has contributions from wing, elevator and propeller induced air flow as given in (22). In (22), $C_{Lw}$, $C_{L\delta_{e}}$ and $C_{Lt}$ are coefficient of lift contributed by wing, elevator and propeller air flow, respectively. The coefficient $C_{Lt}$ is scaled for a maximum propeller RPM value of 12,000. The coefficients of $C_{L\delta_{e}}$ are different for upward and downward elevator deflection ($\delta_{e}>=0$) and upward elevator deflection ($\delta_{e}<0$) respectively. The coefficients $C_{Lw}$, $C_{L\delta_{e}}$ and $C_{Lt}$ are given in Table 6.

(22) $$\begin{array}{c} C_{L}=C_{Lw}+C_{L\delta_{e}}\delta_{e}+C_{Lt}\left(\frac{RPM}{12000}\right) \end{array}$$

The relation between coefficient of drag ($C_{D}$) and total drag force is given in (23).

(23) $$D=C_{D}\left(0.5\rho V_{a}^{2}S\right)$$

**Figure 9:**
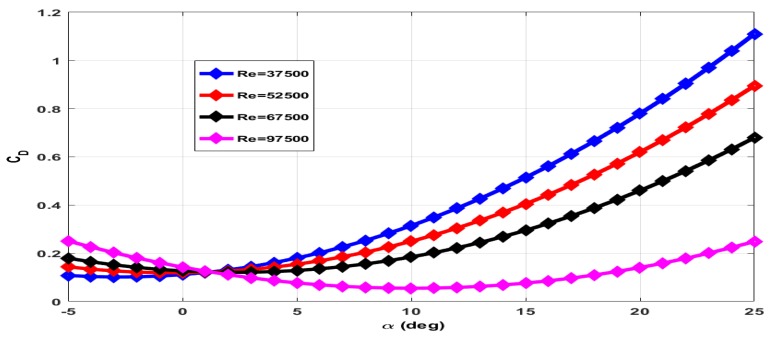
Coefficient of drag at 0${}^{\circ }$ elevator deflection and zero propeller RPM.

**Figure 10:**
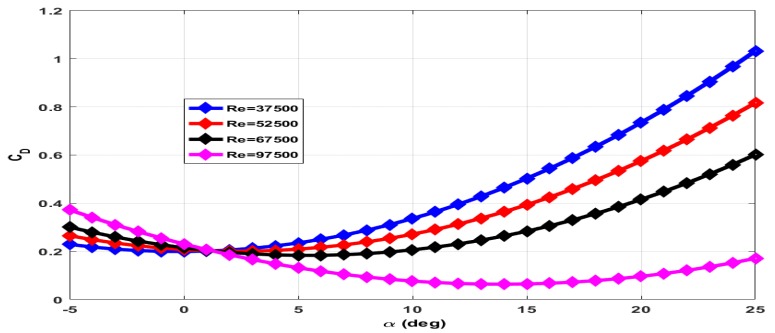
Coefficient of drag at −25${}^{\circ }$ elevator deflection and zero propeller RPM.

**Figure 11:**
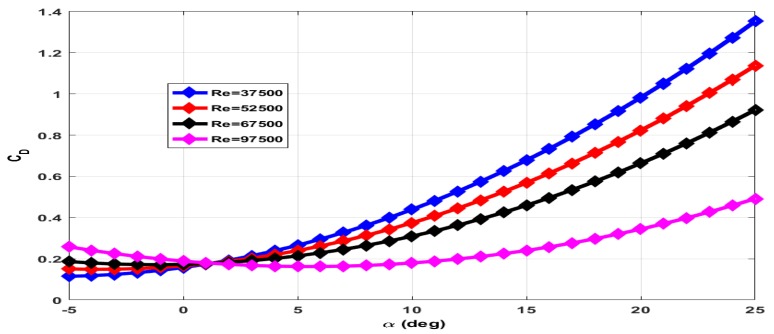
Coefficient of drag at +15${}^{\circ }$ elevator deflection and zero propeller RPM.

The coefficient of drag ($C_{D}$) has contributions of wing ($C_{Dw}$), elevator ($C_{D\delta_{e}}$) and due to propeller wake ($C_{Dt}$). The total axial force measured with rotating propeller includes drag force and thrust force. The contribution of wing and elevator to drag force is measured with the propeller in a stationary condition. The contribution of propeller wake to drag force is estimated through empirical relations. The plot of $C_{D}$ for zero elevator deflection and the stationary propeller is given in Figure 9. The total drag force increases with increase in Reynolds number. However, $C_{D}$ decreases with increase in Reynolds number as $C_{D}\propto \frac{1}{V_{a}^{2}}$ for a given drag force. The plot of $C_{D}$ for −25${}^{\circ }$ elevator deflection and +15${}^{\circ }$ elevator deflection with the stationary propeller is given in Figure 10 and Figure 11 respectively.

The coefficient of drag of the MAV is given in (24). The coefficient of drag due to wing ($C_{Dw}$) is modeled as a function of airspeed, and angle of attack. The expression for $C_{Dw}$ is given in (25) and for $C_{D\delta_{e}}$ is given in (26). The coefficients of $C_{Dw}$ and $C_{D\delta_{e}}$ are given in Table 7 for positive and negative elevator deflection.

(24) $$C_{D}=C_{Dw}+C_{D\delta_{e}}\delta_{e}+C_{Dt}$$

(25) $$C_{Dw}=\left(C_{D1}V_{a}+C_{D01}\right)\alpha^{2}+\left(C_{D2}V_{a}+C_{D02}\right)\alpha +\left(C_{D3}V_{a}+C_{D03}\right)$$

(26) $$C_{D\delta_{e}}=C_{D4}\alpha +C_{D04}$$

The contribution of propeller wake to drag, $C_{Dt}$ is taken from [23] and is given in (27).

(27) $$\begin{array}{c} C_{Dt}=\frac{1.1S_{prop}}{S}+\frac{C_{Lt}C_{L}\left(\frac{RPM}{12000}\right)}{\pi (AR)} \end{array}$$

where $S_{prop}$ is the area swept by one propeller rotation. While taking the measurements with rotating propeller, the total drag force measured is the difference between the MAV drag and the dynamic thrust generated by the propeller. The estimated dynamic thrust, T ($\delta_{th}$) is given in (28).

(28) $$T=\left(0.0989-0.0468\frac{V_{a}}{nd_{p}}\right)\rho n^{2}d_{p}^{4}$$

where *n* is the propeller rotation per second (RPS) and $d_{p}$ is the propeller diameter.

The relation between the coefficient of pitching moment $C_{m}$ and pitching moment (*M*) is given in (29).

(29) $$M=C_{m}\left(0.5\rho V_{a}^{2}S\bar{c}\right)$$

where

(30) $$C_{m}=C_{mw}+C_{m\delta_{e}}\delta_{e}+C_{mt}\left(\frac{RPM}{12000}\right)$$

In (30), $c_{mw}$ is due to wing (31), $C_{m\delta_{e}}$ due to propeller (32) and $C_{mt}$ due to propeller wake (33).

(31) $$C_{mw}=\left(0.1068V_{a}-1.62\right)\alpha +\left(0.0111V_{a}+0.0272\right)$$

(32) $$C_{m\delta_{e}}=-2.1278\alpha -0.3174$$

(33) $$C_{mt}=-0.0448\alpha -0.1791$$

The relation between side force coefficient ($C_{y}$) and side force (*Y*) is given in (34).

(34) $$Y=C_{y}\left(0.5\rho V_{a}^{2}S\right)$$

$C_{y}$ is expressed as a function of sideslip angle (β) and rudder deflection ($\delta_{r}$) and is given in (35).

(35) $$C_{y}=C_{y\beta }\beta +C_{y\delta_{r}}\delta_{r}$$

The derivatives $C_{y\beta }$ and $C_{y\delta_{r}}$ are given in (36) and (37) respectively.

(36) $$\begin{array}{c} C_{y\beta }=-1.3823-1.1058\left(\frac{RPM}{12000}\right) \end{array}$$

and

(37) $$C_{y\delta_{r}}=-0.4345-0.3476\left(\frac{RPM}{12000}\right)$$

The other static derivatives $C_{l\beta }$, $C_{n\beta }$ and control derivatives $C_{l\delta_{r}}$, $C_{n\delta_{r}}$ are obtained through empirical relations given in [20].

### 2.2. Estimation of Dynamic Derivatives

Estimation of dynamic derivatives through wind tunnel tests requires sophisticated instruments like rotating rigs. The wind tunnel does not have the required instrumentation for measuring dynamic derivatives. So the empirical relations provided in [20] is used here for estimating the dynamic derivatives. The dynamic derivatives are a function of α, $V_{a}$, β and the MAV configuration parameters like specifications of the vertical tail, winglets etc. The estimated static derivatives from wind tunnel tests and dynamic derivatives from empirical relation is given for a velocity of 8 m/s, α=13.1${}^{\circ }$ and β=−3.05${}^{\circ }$ is shown in Table 8. This corresponds to trim conditions as explained in the next section.

## 3. MAV Flight Envelope and Trim

The MAV is said to be flying in a trimmed condition when the net forces and moments acting on MAV equals to zero. The upper limit on velocity in the flight envelope of the MAV is determined by the thrust available ($T_{a}$) and thrust required for different flight velocities. The lower value of velocity is determined by the maximum safe operating angle of attack. Flying at an angle of attack near stall value is not recommendable as a small disturbance may lead to a stall. From the wind tunnel testing, it is observed that $\alpha_{stall}$ of the MAV is higher than 25${}^{\circ }$. Hence a maximum angle of attack of 25${}^{\circ }$ is taken as the limit for normal flying conditions. The trim conditions for straight and constant altitude flight are obtained by equating $\dot{u}=0$, $\dot{v}=0$, $\dot{w}=0$, $\dot{p}=0$, $\dot{q}=0$, $\dot{r}=0$, $\dot{\phi }=0$, $\dot{\theta }=0$ and $\dot{z}=0$. The equations are solved using the *fsolve* routine in MATLAB. The trim conditions for straight and constant altitude flight are given in Table 9. As seen in Table 9, at $V_{a}$= 6 m/s, cruise α is slightly lower than 25${}^{\circ }$. At $V_{a}$= 13 m/s, thrust required is almost equals thrust available. So the flight envelope of MAV is determined as in the velocity range of 6–13 m/s.

Note that unlike bigger aircraft, the value of β, ϕ and $\delta_{r}$ is non-zero for MAV for straight and constant altitude flight conditions. This is due to the counter torque exerted by the rotating propeller.The counter torque cannot be neglected for MAVs as the inertia $J_{xx}$ is small when compared to bigger UAV. The effects of counter torque on the dynamics of a small fixed wing MAV is explained in [24].

## 4. Linear State Space Model of MAV

The linear state space model is computed by linearzing the nonlinear dynamic model equations of the MAV about trim conditions. The variables used in this section are linearized, for example $\tilde{u}$ denotes the linearized variable for *u* and so on. The linear state space model equations can be separated into linear longitudinal state space model and linear lateral state space model [25]. The linear longitudinal state space model is given in (38).

(38) $${\dot{X}}_{long}=A_{long}X_{long}+B_{long}U_{long}$$

where $X_{long}={\left[\tilde{u},\;\;\tilde{w},\;\;\tilde{q},\;\;\tilde{\theta }\right]}^{T}$ and $U_{long}={\left[{\tilde{\delta }}_{e},\;\;{\tilde{\delta }}_{t}\right]}^{T}$. The matrices $A_{long}$ and $B_{long}$ is given in (39) and (40) respectively, for a trim condition corresponding to $V_{a}=8$ m/s.

(39) $$A_{long}=\left(\begin{array}{cccc} -0.2317 & -0.0271 & -1.7507 & -9.5507\\ -1.0715 & -5.4429 & 7.5206 & -2.2393\\ 64.6310 & -147.4623 & -12.9852 & 0\\ 0 & 0 & 1.0 & 0 \end{array}\right)$$

(40) $$B_{long}=\left(\begin{array}{cc} 3.9609 & 0.2061\\ -6.4834 & 0\\ -677.4730 & 0\\ 0 & 0 \end{array}\right)$$

The phugoid mode natural frequency ($\omega_{ph}$) and damping ratio ($\zeta_{ph}$) is 1.94 rad/s and 0.283 respectively. The short period mode natural frequency ($\omega_{sp}$) and damping ratio ($\zeta_{sp}$) is 35.7 rad/s and 0.246 respectively. The variation of short period mode and phugoid mode natural frequency and damping ratio with respect to flight velocity is shown in Table 10. The $\omega_{sp}$ increases with increase in velocity where as $\omega_{ph}$ decreases. The $\zeta_{sp}$ increases initially with increase in velocity, but later decreases. Whereas, $\zeta_{ph}$ decreases initially with increase in velocity, but later increases. The transfer functions $\frac{\tilde{u}\left(s\right)}{{\tilde{\delta }}_{e}\left(s\right)}$, $\frac{\tilde{w}\left(s\right)}{{\tilde{\delta }}_{e}\left(s\right)}$, $\frac{\tilde{q}\left(s\right)}{{\tilde{\delta }}_{e}\left(s\right)}$, $\frac{\tilde{\theta }\left(s\right)}{{\tilde{\delta }}_{e}\left(s\right)}$, $\frac{\tilde{u}\left(s\right)}{{\tilde{\delta }}_{th}\left(s\right)}$, $\frac{\tilde{w}\left(s\right)}{{\tilde{\delta }}_{th}\left(s\right)}$, $\frac{\tilde{q}\left(s\right)}{{\tilde{\delta }}_{th}\left(s\right)}$, $\frac{\tilde{\theta }\left(s\right)}{{\tilde{\delta }}_{th}\left(s\right)}$ for $V_{a}=8$ m/s are given in (41) to (48).

(41) $$\frac{\tilde{u}\left(s\right)}{{\tilde{\delta }}_{e}\left(s\right)}=\frac{3.96(s+304.7)(s+11.5)(s+1.78)}{\Delta_{long}\left(s\right)}$$

(42) $$\frac{\tilde{w}\left(s\right)}{{\tilde{\delta }}_{e}\left(s\right)}=\frac{-6.4\left(s+800.1\right)\left(s^{2}-0.038s+1.461\right)}{\Delta_{long}\left(s\right)}$$

(43) $$\frac{\tilde{q}\left(s\right)}{{\tilde{\delta }}_{e}\left(s\right)}=\frac{-677.5s(s+4.36)(s-0.48)}{\Delta_{long}\left(s\right)}$$

(44) $$\frac{\tilde{\theta }\left(s\right)}{{\tilde{\delta }}_{e}\left(s\right)}=\frac{-677.5(s+4.36)(s-0.48)}{\Delta_{long}\left(s\right)}$$

(45) $$\frac{\tilde{u}\left(s\right)}{{\tilde{\delta }}_{th}\left(s\right)}=\frac{0.21\left(s-0.28\right)\left(s^{2}+18.7s+1815\right)}{\Delta_{long}\left(s\right)}$$

(46) $$\frac{\tilde{w}\left(s\right)}{{\tilde{\delta }}_{th}\left(s\right)}=\frac{-0.22(s-440.3)(s-0.31)}{\Delta_{long}\left(s\right)}$$

(47) $$\frac{\tilde{q}\left(s\right)}{{\tilde{\delta }}_{e}\left(s\right)}=\frac{13.3s(s+7.89)}{\Delta_{long}\left(s\right)}$$

(48) $$\frac{\tilde{\theta }\left(s\right)}{{\tilde{\delta }}_{th}\left(s\right)}=\frac{13.3(s+7.89)}{\Delta_{long}\left(s\right)}$$

In (41) to (48), $\Delta_{long}\left(s\right)$ is the characteristic polynomial for linear longitudinal dynamics and is given in (49).

(49) $$\Delta_{long}\left(s\right)=\left(s^{2}+17.56s+1274.5\right)\left(s^{2}+1.098s+3.764\right)$$

The linear lateral state space model is given in (50).

(50) $${\dot{X}}_{lat}=A_{lat}X_{lat}+B_{lat}U_{lat}$$

where $X_{lat}={\left[\tilde{v},\;\;\tilde{p},\;\;\tilde{r},\;\;\tilde{\phi }\right]}^{T}$ and $U_{lat}=\left[{\tilde{\delta }}_{r}\right]$. The matrices $A_{lat}$ and $B_{lat}$ corresponding to trim conditions of $V_{a}=8$ m/s is given in (51) and (52).

(51) $$A_{lat}=\left(\begin{array}{cccc} -3.1260 & 1.7637 & -7.6035 & 9.5445\\ -41.3432 & -2.1395 & 2.2497 & 0\\ 217.5970 & -2.7395 & -23.3369 & 0\\ 0 & 1.0000 & 0.2345 & 0 \end{array}\right)$$

(52) $$B_{lat}=\left(\begin{array}{c} -7.8605\\ 53.7014\\ 740.5201\\ 0 \end{array}\right)$$

The lateral dynamics consists of Dutch roll mode, roll subsidence mode and spiral mode. The variation of Dutch roll mode natural frequency ($\omega_{dr}$) and damping ratio ($\zeta_{dr}$) is given in Table 11. Similar to $\omega_{sp}$, $\omega_{dr}$ also increases with increase in flight velocity. The damping ratio, $\zeta_{dr}$ decreases with increase in velocity. The roll subsidence mode pole ($P_{rl}$) remains nearly constant as shown in Table 11. The spiral mode pole, ($P_{sl}$) is stable for all flight velocity. The transfer functions $\frac{\tilde{v}\left(s\right)}{{\tilde{\delta }}_{r}\left(s\right)}$, $\frac{\tilde{p}\left(s\right)}{{\tilde{\delta }}_{r}\left(s\right)}$, $\frac{\tilde{r}\left(s\right)}{{\tilde{\delta }}_{r}\left(s\right)}$, $\frac{\tilde{\phi }\left(s\right)}{{\tilde{\delta }}_{r}\left(s\right)}$ is given in Equations (53) to (56) for $V_{a}=8$ m/s.

(53) $$\frac{\tilde{v}\left(s\right)}{{\tilde{\delta }}_{r}\left(s\right)}=\frac{-7.86(s+729)(s+2.7)(s-1.998)}{\Delta_{lat}\left(s\right)}$$

(54) $$\frac{\tilde{p}\left(s\right)}{{\tilde{\delta }}_{r}\left(s\right)}=\frac{53.7\left(s^{2}+63.8s+6246.3\right)\left(s-0.282\right)}{\Delta_{lat}\left(s\right)}$$

(55) $$\frac{\tilde{r}\left(s\right)}{{\tilde{\delta }}_{r}\left(s\right)}=\frac{740.5\left(s^{2}-2.14s+111.19\right)\left(s+4.90\right)}{\Delta_{lat}\left(s\right)}$$

(56) $$\frac{\tilde{\phi }\left(s\right)}{{\tilde{\delta }}_{r}\left(s\right)}=\frac{227.3(s^{2}+17.1s+1547.6)}{\Delta_{lat}\left(s\right)}$$

In (53) to (56), $\Delta_{lat}\left(s\right)$ is the characteristic polynomial for linear lateral dynamics and is given in (57).

(57) $$\Delta_{lat}\left(s\right)=\left(s^{2}+25.64s+1785.6\right)\left(s+2.08\right)\left(s+0.871\right)$$

## 5. Closed Loop Flight Test Results

A static output feedback controller is designed for improving the damping ratios of short period mode, phugoid mode and Dutch roll mode. The controller synthesis methodology is similar to the algorithm explained in [26]. The controller is synthesized in discrete time domain with a sampling time of 20 ms. The MAV linear state space model corresponding to a velocity of 8 m/s is used as the plant model. The structure of the controller is given by

(58) $$U_{c}\left(k\right)=FY\left(k\right)$$

where $U_{c}\left(k\right)={\left[{\tilde{\delta }}_{e}\left(k\right),\;\;{\tilde{\delta }}_{th}\left(k\right)\right]}^{T}$, $Y\left(k\right)={\left[\tilde{q}\left(k\right),\;\;\tilde{\theta }\left(k\right)\right]}^{T}$ for longitudinal control and *F* is a constant feedback gain matrix. For the control of lateral dynamics, $U_{c}\left(k\right)=\left[{\tilde{\delta }}_{r}\left(k\right)\right]$ and $Y\left(k\right)={\left[\tilde{p}\left(k\right),\;\;\tilde{r}\left(k\right),\;\;\tilde{\phi }\left(k\right)\right]}^{T}$. The procedure adopted in controller synthesis is explained below.

Let the state space model of the open loop plant be given as below.

(59) $$\dot{X}=AX+BU_{c}$$

(60) Y=CX

The control design objective is to find a static output feedback controller of the form given in (58) that minimizes the $H_{\infty }$ norm of the following mixed sensitivity function.

(61) $${∥T_{zw_{d}}∥}_{\infty }={∥\left(\begin{array}{c} W_{1}S\\ W_{2}T\\ W_{3}KS \end{array}\right)∥}_{\infty }$$

In (61), $T_{zw_{d}}$ represents the closed loop transfer function from disturbance input to performance output. The sensitivity function, complementary sensitivity function and control input sensitivity function is denoted by *S*, *T* and KS respectively. The open loop plant given in (59) and (60) is augmented with frequency dependent weighting functions $W_{1}$, $W_{2}$ and $W_{3}$ to obtain the generalized plant. The discrete time equivalent of the closed loop generalized plant is given in (62)–(65).

(62) $$X_{d}\left(k+1\right)=\left(A_{d}+B_{d}FC_{d}\right)X_{d}\left(k\right)+B_{d}W_{d}\left(k\right)$$

(63) $$Z_{1}\left(k\right)=\left(C_{1d}+D_{11d}FC_{d}\right)X_{d}\left(k\right)+D_{12d}W_{d}\left(k\right)$$

(64) $$Z_{2}\left(k\right)=\left(C_{2d}+D_{21d}FC_{d}\right)X_{d}\left(k\right)+D_{22d}W_{d}\left(k\right)$$

(65) $$Z_{3}\left(k\right)=\left(C_{3d}+D_{31d}FC_{d}\right)X_{d}\left(k\right)+D_{32d}W_{d}\left(k\right)$$

In (63), $Z_{1}\left(k\right)$ is the output of weighted sensitivity function $\left(W_{1}S\right)$. Similarly, $Z_{2}\left(k\right)$ and $Z_{3}\left(k\right)$ given in (64) and (65) are the outputs of weighted complimentary sensitivity function $\left(W_{2}T\right)$ and weighted control input sensitivity function $\left(W_{3}KS\right)$ respectively. The disturbance input to the plant is denoted by $W_{d}$. The controller is synthesized by solving the following linear matrix inequality (LMI) stated in (66), [27].

(66) $$\begin{array}{c} \left(\begin{array}{cc} -\mu P & -{\left(A_{d}+B_{ud}FC_{d}\right)}^{T}R^{T}\\ -R(A_{d}+B_{ud}FC_{d}) & \mu P-R-R^{T} \end{array}\right)<0 \end{array}$$

In (66), μ<1 is the radius of the circle in discrete time complex plane, inside which poles of the closed loop system need to placed. The unknowns are the matrix P>0 and feedback gain matrix *F*. The elements of the matrix *R* are selected initially to solve for *P* and *F*. The elements of the matrix *R* are the input variables of genetic algorithm (GA), that minimizes the performance index given in (61). The feedback gain matrix is given in (67) for control of longitudinal dynamics and in (68) for control of lateral dynamics respectively. Please note that the gain from *q* and θ to $\delta_{th}$ is high when compared to $\delta_{e}$. This is because $\delta_{e}$ falls in a range of −0.2618 radians (−15${}^{\circ }$) to 0.4363 radians (+25${}^{\circ }$), where as $\delta_{th}$ falls between 0–120 RPS (rotations per second).

(67) $$F_{long}=\left[\begin{array}{cc} 0.0253 & -0.3430\\ -15.3382 & 5.5186 \end{array}\right]$$

(68) $$F_{lat}=\left[\begin{array}{ccc} -0.0189 & -0.0212 & -0.0240 \end{array}\right]$$

A comparison between open loop and closed loop damping ratios and natural frequencies of short period mode and phugoid mode for continuous time system are given in Table 12. The short period mode damping ratio is increased from open loop system value of 0.246 to 0.509 for closed loop system. The short period mode frequency of closed loop system is slightly increased when compared to open loop system. The damping ratio $\zeta_{sp}$ is improved by feedback of *q* to $\delta_{e}$. Due to weight and power restrictions in MAV, a low bandwidth actuator is used for elevator and rudder control surfaces. So a very high $\zeta_{sp}$ is not practically realizable. The phugoid mode frequency remains almost same for open loop and closed loop system. There is a slight improvement in $\zeta_{ph}$ for closed loop system. For achieving a high $\zeta_{ph}$, velocity to $\delta_{th}$ feedback is required. Since MAV does not have any light weight and accurate airspeed sensor, a very high $\zeta_{ph}$ is not achievable.

The open loop and closed loop modes of lateral dynamics for continuous time system are given in Table 13. The Duthch roll mode damping ratio is improved for closed loop system. The $\zeta_{dr}$ is improved by *r* feedback to $\delta_{r}$. Due to limitations in actuator bandwidth, a very high $\zeta_{dr}$ is not practically feasible.

**Figure 12:**
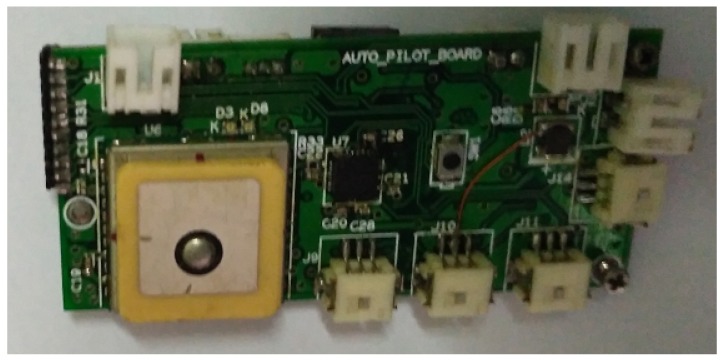
Autopilot hardware.

The flight testing is conducted using a customized 7 g autopilot hardware shown in Figure 12. The autopilot consists of 3-axis accelerometer, 3-axis rate gyro, 3-axis magnetometer, altimeter, GPS and a bi-directional communication module. Servomotors are used for elevator and rudder control surfaces. The thrust is generated by a BLDC motor. The sensors and actuators are interfaced to a 32-bit ARM Cortex-M3 processor, where all the computations are done. Control input is given at a sampling time of 20 ms.

**Figure 13:**
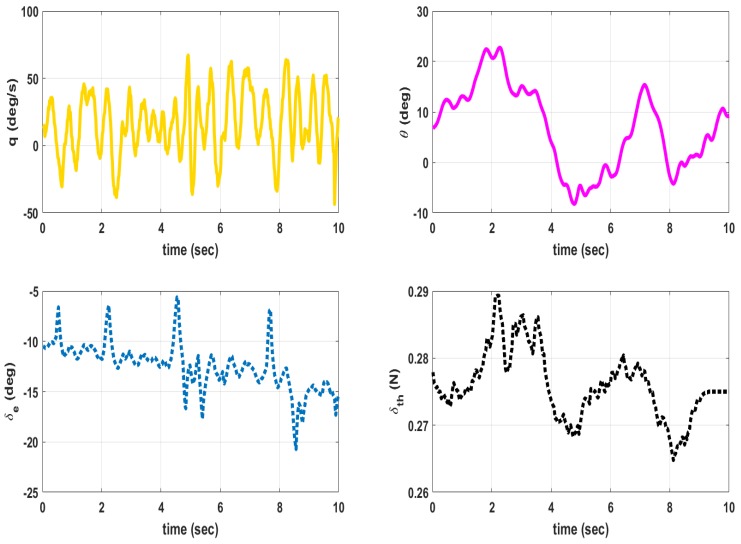
Outputs (q,θ) and inputs ($\delta_{e},\;\;\delta_{th}$) for longitudinal control.

**Figure 14:**
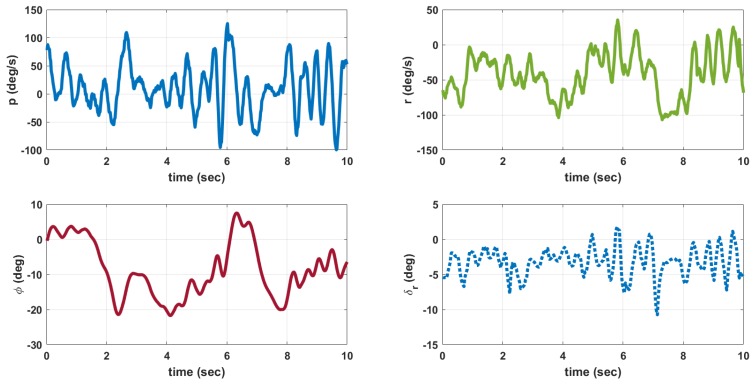
Outputs (p,r,ϕ) and input ($\delta_{r}$) for lateral control.

**Figure 15:**
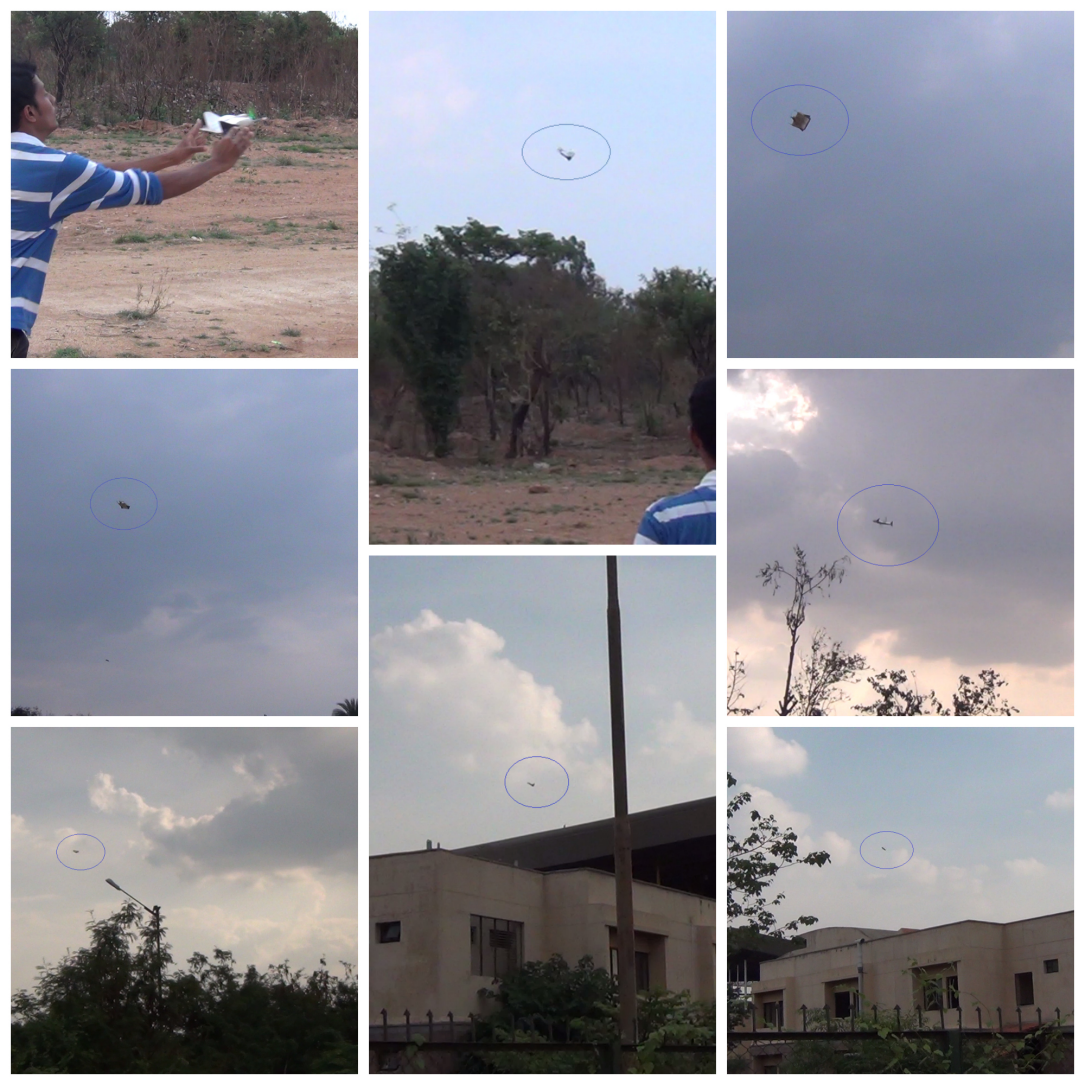
Photograph showing MAV flight.

The MAV is hand launched at a trim condition corresponding to $V_{a}$ = 8 m/s as in Table 9. Apart from the feedback control inputs, the inputs from the pilot are also given. The flight tests are conducted under mild wind conditions to evaluate the flight stability characteristics. The plot of outputs *q*, θ and inputs $\delta_{e}$, $\delta_{th}$ for longitudinal control are shown in Figure 13. When $\delta_{th}$ is high close to 2 seconds, the MAV exhibits pitch-up motion as seen from θ plot. The outputs *p*, *r*, ϕ and input $\delta_{r}$ for lateral control is given in Figure 14. The magnitude of *p* is higher than *q* and *r* due to counter torque and cross wind effects. The control inputs shown in Figure 13 and Figure 14 are combined input from pilot and feedback control. The inputs from feedback control and those from pilot are not recorded separately. A photograph of MAV flight is shown in Figure 15. The flight test indicate that the static output feedback controller designed based on the longitudinal and lateral model developed in this paper stabilizes the MAV flight.

### Comparison between Predicted p, q, r Data from Model and Flight Test

**Figure 16:**
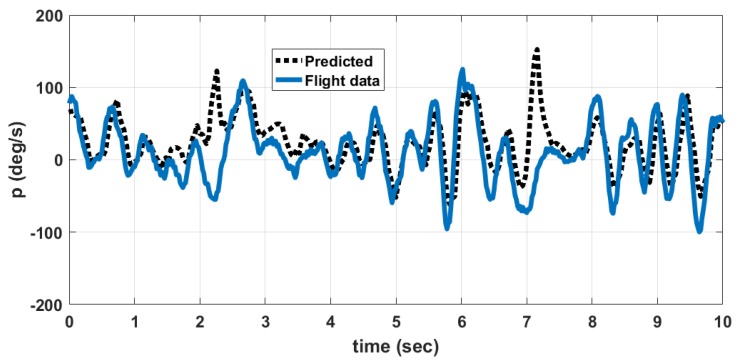
Comparison of angular rate *p*, predicted by model and flight data.

**Figure 17:**
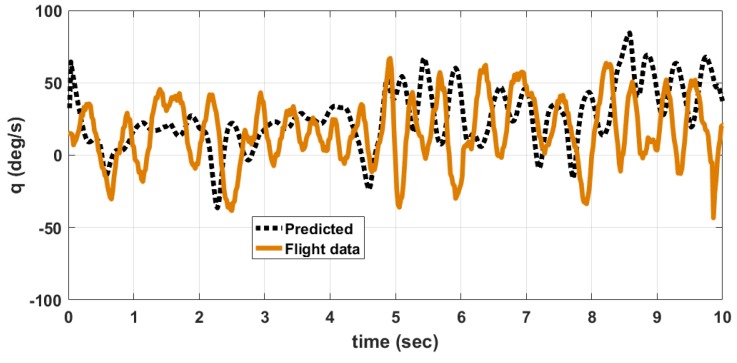
Comparison of angular rate *q*, predicted by model and flight data.

**Figure 18:**
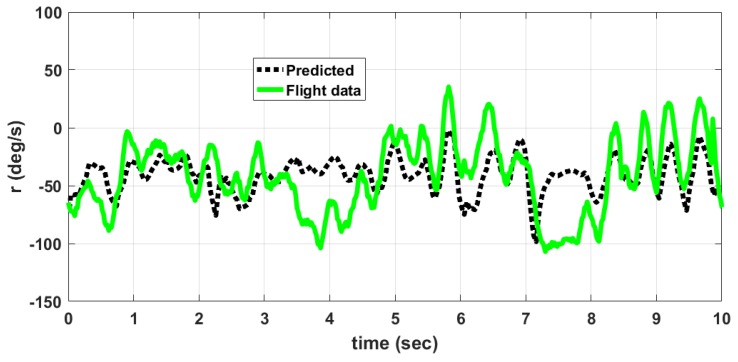
Comparison of angular rate *r*, predicted by model and flight data.

A comparison between data obtained from flight tests and those predicted by the developed nonlinear model for the angular rates *p*, *q*, *r* is shown in Figure 16, Figure 17 and Figure 18 respectively. The predicted data is obtained by giving the elevator, rudder and thrust inputs shown in Figure 13 and Figure 14, to the nonlinear equations given in (1) to (18). The discrepancy between flight data and predicted data is due to the following reasons. (1) The wind disturbances acting on the MAV during flight is not measured. So the response of MAV due to wind disturbances cannot be predicted. (2) Due to manufacturing errors, the MAV used for flight testing is not exactly identical to the one used for wind tunnel testing.

The error between flight data and predicted data is given in Table 14. The percentage error $P_{E}\left(v\right)$ is computed using (69).

(69) $$\begin{array}{c} P_{E}\left(v\right)=\frac{A(|v_{p}-v_{f_{d}}|)}{M(|v_{f_{d}}|)}\times 100\% \end{array}$$

where A(.) denotes the average, $v_{p}$ denotes the predicted value of variable (*p*, *q*, *r*), $v_{f_{d}}$ denotes the flight data of the same variable and M(.) denotes the maximum. The angular rate *q* depends upon elevator input and thrust input. The angular rate *p* depends upon rudder input and counter torque generated due to propeller rotation. The angular rate rate *r* depends upon mainly on rudder input. So the prediction error is higher for *p* and *q* when compared to *r*. This is also due to the fact that the thrust and counter torque model used in the nonlinear model is determined at constant voltage of 8.4 V input to the motor. However, in actual flight, the battery voltage is varying and is not measured. This accounts for an error in predicting dynamic thrust and counter torque during flight.

## 6. Conclusions

This paper presented the nonlinear 6DOF dynamic modeling of a 150 mm wingspan fixed wing MAV. The effects of propeller wake on lift, drag and pitching moment are quantified. There is an increase of 10% in lift considering the propeller effects. The propeller wake had increased drag substantially. The longitudinal static stability improves with propeller effects. The dynamic thrust is also estimated from wind tunnel test results.

Unlike a bigger aircraft, the value of sideslip angle, roll angle and rudder deflection is non-zero to maintain a straight and constant altitude flight conditions. This is due to the effect of significant counter torque generated by the rotating propeller. The linear longitudinal and lateral state space models are presented. The $H_{\infty }$ static output feedback controller improves the damping ratios of various modes of longitudinal and lateral dynamics. The closed loop flight test results with the static output feedback controller indicate satisfactory stability characteristics of the MAV flight. The obtained flight data is compared with the flight data generated from the nonlinear model to verify the modeling accuracy.

## Figures and Tables

**Table 1:** 150 mm MAV—specifications.

Attribute	Value
Planform	Rectangular
Airfoil	E387
Weight (grams)	53
Velocity, $V_{a}$ (m/s)	6–13
Chord, $\bar{c}$ (m)	0.11
Span, *b* (m)	0.15
Control surfaces	Elevator and rudder
$J_{xx}\;\left(Kgm^{2}\right)$	$1182.68\times 10^{-7}$
$J_{yy}\;\left(Kgm^{2}\right)$	$773.56\times 10^{-7}$
$J_{zz}\;\left(Kgm^{2}\right)$	$431.24\times 10^{-7}$
$J_{xz}\;\left(Kgm^{2}\right)$	$42.49\times 10^{-7}$

**Table 2:** Airfoil specifications.

Attribute	Value
thickness to chord, t/c	25%
Maximum thickness position	31% from leading edge
Maximum camber	3.8%
Maximum camber position	40% from leading edge

**Table 3:** Wind tunnel specifications.

Attribute	Value
Type	Open circuit
Contraction ratio	9:1
Test section size	0.8 m × 1.2 m × 2.5 m
Total length	17 m
Velocity range	1 m/s–45 m/s
Turbulence intensity	<0.1%

**Table 4:** Propeller specifications.

Attribute	Value
Diameter	12.7 cm
Pitch	7.6 cm
Chord max.	1.58 cm
Twist max.	20${}^{\circ }$

**Table 5:** Propeller static thrust with AP05 motor.

RPM	Static Thrust (N)
10,800	0.394
12,800	0.553
15,500	0.826

**Table 6:** Coefficient of lift data.

Velocity (m/s)	$C_{\mathrm{Lw}}$	$C_{L\delta_{e}}$, $\delta_{e}>=0$	$C_{L\delta_{e}}$, $\delta_{e}<0$	$C_{\mathrm{Lt}}$
5	3.3α + 0.057	0.7792α + 1.3751	0.1299α + 1.9863	0.1493α − 0.0177
7	2.3α + 0.22	0.55α + 0.7334	0.9549α + 1.4133	2.0153α − 0.0318
9	1.6α + 0.24	0.7105α + 1.2223	0.9167α + 1.2223	1.2308α − 0.0154
13	2.2α + 0.34	0.0142α + 0.7105	0.9150α + 1.2210	0.3014α + 0.0125

**Table 7:** Coefficient of drag data.

Coefficient	$\delta_{e}>=0$	$\delta_{e}<0$
$C_{D1}$	−0.1816	−0.1816
$C_{D01}$	5.2074	5.2074
$C_{D2}$	−0.1764	1.2947
$C_{D02}$	−0.1764	1.2947
$C_{D3}$	0.0039	0.0919
$C_{D03}$	0.0039	0.0919
$C_{D4}$	1.7189	0.3401
$C_{D04}$	0.8709	−0.2017

**Table 8:** Longitudinal and Lateral Dynamic Derivatives.

Roll Rate Derivatives	Pitch Rate Derivatives	Yaw Rate Derivatives
$C_{yp}=-0.46$	$C_{Lq}=3.53$	$C_{yr}=1.71$
$C_{lp}=-0.31$	$C_{Dq}=0.0$	$C_{lr}=0.34$
$C_{np}=-0.14$	$C_{mq}=-2.27$	$C_{nr}=-1.22$

**Table 9:** Trim conditions of MAV.

Velocity (m/s)	α(deg)	β(deg)	ϕ(deg)	θ(deg)	$\delta_{e}\left(\deg\right)$	$\delta_{r}\left(\deg\right)$	$\delta_{\mathrm{th}}\left(N\right)$	$T_{a}\left(N\right)$
6	23.65	−6.41	−2.39	23.88	−23.06	15.76	0.3213	0.4018
7	15.93	−3.70	−1.85	16.04	−17.65	8.83	0.2642	0.3909
8	13.10	−3.05	−2.07	13.20	−14.85	7.19	0.2312	0.3804
9	12.46	−2.56	−2.21	12.55	−12.14	6.03	0.2211	0.3692
10	9.79	−1.98	−2.11	9.86	−8.16	4.61	0.1873	0.3583
11	7.03	−1.52	−1.98	7.08	−3.74	3.51	0.1557	0.3474
12	2.04	−1.37	2.29	2.09	0.77	3.08	0.1896	0.3365
13	−0.782	−1.42	−3.09	−0.705	1.012	3.15	0.2873	0.3257

**Table 10:** Variation of natural frequencies and damping ratio of longitudinal modes of MAV.

Velocity (m/s)	$\omega_{\mathrm{sp}}$ (rad/s)	$\zeta_{\mathrm{sp}}$	$\omega_{\mathrm{ph}}$ (rad/s)	$\zeta_{\mathrm{ph}}$
6	27.1	0.17	2.34	0.481
7	31.7	0.251	2.15	0.284
8	35.7	0.246	1.94	0.283
9	39.3	0.2196	1.77	0.24
10	43.2	0.213	1.72	0.146
11	47.2	0.210	1.61	0.069
12	51.2	0.213	1.39	0.162
13	53.6	0.183	1.25	0.355

**Table 11:** Variation of natural frequencies and damping ratio of lateral modes of MAV.

Velocity (m/s)	$\omega_{\mathrm{dr}}$ (rad/s)	$\zeta_{\mathrm{dr}}$	$P_{\mathrm{rl}}$ (rad/s)	$P_{\mathrm{sl}}$ (rad/s)
6	32.36	0.468	−1.99	−1.351
7	36.8	0.382	−1.52	−1.081
8	42.3	0.303	−2.08	−0.871
9	47.4	0.263	−2.52	−0.592
10	51.9	0.238	−2.72	−0.676
11	56.0	0.221	−2.78	−0.792
12	60.6	0.214	−2.36	−1.323
13	66.6	0.213	−2.15	−1.667

**Table 12:** Open loop and closed loop longitudinal modes of MAV.

Mode	Open Loop	Closed Loop
Short period, $\omega_{sp}$ (rad/s)	35.7	39.9
Short period, $\zeta_{sp}$	0.246	0.509
Phugoid, $\omega_{ph}$ (rad/s)	1.94	1.92
Phugoid, $\zeta_{ph}$	0.283	0.394

**Table 13:** Open loop and closed loop lateral modes of MAV.

Mode	Open Loop	Closed Loop
Dutch roll, $\omega_{dr}$ (rad/s)	42.3	44.6
Dutch roll, $\zeta_{dr}$	0.303	0.502
Roll subsidence, $P_{rl}$ (rad/s)	−2.08	−6.13
Spiral, $P_{sl}$ (rad/s)	−0.871	−1.90

**Table 14:** Percentage error in prediction.

Variable	Value
$P_{E}\left(p\right)$	21.13%
$P_{E}\left(q\right)$	31.74%
$P_{E}\left(r\right)$	13.57%

## Supplementary Materials

The flight video is available at https://www.youtube.com/watch?v=SVGpc5XLyKEfeature=youtu.be.

## Abbreviations

The following abbreviations are used in this manuscript:

BLDC	Brushless direct current
CFD	Computational fluid dynamic
DOF	Degree of freedom
GPS	Global positioning system
MAV	Micro air vehicle
RPM	Rotations per minute
RPS	Rotations per second
UAV	Unmanned air vehicle

## Appendix A

$C_{L}$—Coefficient of lift

$C_{D}$—Coefficient of drag

$C_{m}$—Pitching moment coefficient of the wing

$C_{Lq}$—Coefficient for change in lift with respect to *q*

$C_{Dq}$—Coefficient for change in drag with respect to *q*

$C_{y}$—Side force coefficient

$C_{yp}$—Coefficient for change in side force with respect to *p*

$C_{yr}$—Coefficient for change in side force with respect to *r*

$C_{y\beta }$—Slope of the side force coefficient of MAV versus β curve

$C_{y\delta_{r}}$—Slope of the side force coefficient of MAV versus $\delta_{r}$ curve

$C_{mq}$—Coefficient for change in pitching moment with respect to *q*

$C_{lp}$—Coefficient for change in rolling moment with respect to *p*

$C_{lr}$—Coefficient for change in rolling moment with respect to *r*

$C_{l\beta }$—Coefficient for change in rolling moment with respect to β

$C_{l\delta_{r}}$—Slope of the rolling moment coefficient of MAV versus $\delta_{r}$ curve

$C_{np}$—Coefficient for change in yawing moment with respect to *p*

$C_{nr}$—Coefficient for change in yawing moment with respect to *r*

$C_{n\beta }$—Coefficient for change in yawing moment with respect to β

$C_{n\delta_{r}}$—Coefficient for change in yawing moment with respect to $\delta_{r}$

## References

[1] Pines D.J., Bohorquez F. (2006). Challenges facing future micro air vehicle development. J. Aircr..

[2] Petricca L., Ohlckers P., Grinde C. (2011). Micro- and Nano-Air Vehicles: State of the Art. Int. J. Aerosp. Eng..

[3] Grasmeyer J.M., Keennon M.T. (2001). Development of the Black Widow micro air vehicle. AIAA Aerosp. Sci. Meet. Exhib..

[4] Srigrarom S., Chan W.L. (2015). Ornithopter Type Flapping Wings for Autonomous Micro Air Vehicles. Aerospace.

[5] Tran H.K., Chiou J.S. (2016). PSO-Based Algorithm Applied to Quadcopter Micro Air Vehicle Controller Design. Micromachines.

[6] Pappu V.S.R., Steck J.E., Ramamurthi G. (2016). Turbulence Effects on Modified State Observer-Based Adaptive Control: Black Kite Micro Aerial Vehicle. Aerospace.

[7] Null W., Noseck A., Shkarayev S. Effects of Propulsive-Induced Flow on the Aerodynamics of Micro Air Vehicles. Proceedings of the AIAA Applied Aerodynamics Conference.

[8] Gamble B.J., Reeder M.F. (2009). Experimental Analysis of Propeller—Wing Interactions for a Micro Air Vehicle. J. Aircr..

[9] Durai A. (2014). Experimental Investigation of Lift and Drag Characteristics of a Typical MAV under Propeller Induced Flow. Int. J. Micro Air Veh..

[10] Arivoli D., Dodamani R., Antony R., Suraj C.S., Ramesh G., Ahmed S. Experimental Studies on a Propelled Micro Air Vehicle. Proceedings of the 29th AIAA Applied Aerodynamics Conference, No. AIAA 2011-3656.

[11] Sudhakar S., Chandankumar A., Venkatakrishnan L. (2017). Influence of propeller slipstream on vortex flow field over a typical micro air vehicle. Aeronaut. J..

[12] Xiao T., Li Z., Deng S., Ang H., Zhou X. (2016). Numerical study on the flow characteristics of micro air vehicle wings at low Reynolds numbers. Int. J. Micro Air Veh..

[13] Zhan J.X., Wang W.J., Wu Z., Wang J.J. (2006). Wind-Tunnel Experimental Investigation on a Fix-Wing Micro Air Vehicle. J. Aircr..

[14] Mueller T.J., Kellog J.C., Ifju P.G., Shkarayev S.V. (2006). Introduction to the Design of Fixed Wing Micro Air Vehicles—Including Three Case Studies.

[15] Kuo Z.S., Soong C.Y., Chang Y.S. (2010). Dynamic Modeling and Analysis of Fixed-Wing Micro Air Vehicle. Trans. Jpn. Soc. Aerosp. Sci..

[16] Koehl A., Rafaralahy H., Martinez B., Boutayeb M. Modeling and Identification of a Launched Micro Air Vehicle: Design and Experimental Results. Proceedings of the AIAA Modeling and Simulation Technologies Conference, AIAA 2010-8360.

[17] Uhlig D.V., Selig M.S. (2017). Modeling Micro Air Vehicle Aerodynamics in Unsteady High Angle-of-Attack Flight. J. Aircr..

[18] Pushpangathan J.V., Srinivasan K., Bhat M.S. (2014). Design and Development of Fixed-Wing Nano Air Vehicle. J. Unmanned Syst. Technol..

[19] Harikumar K., Dhall S., Bhat M.S. Nonlinear modeling and control of coupled dynamics of a fixed wing micro air vehicle. Proceedings of the Indian Control Conference (ICC).

[20] Roskam J. (1990). Airplane Design, Part 6.

[21] Beard R.W., McLain T.W. (2012). Small Unmanned Aircraft: Theory and Practice.

[22] Deters R.W., Selig M.S. Static Testing of Micro Propellers. Proceedings of the 26th AIAA Applied Aerodynamics Conference, No. AIAA 2008-6246.

[23] Hoener S.F. (1965). Fluid-Dynamic Drag.

[24] Pushpangathan J.V., Bhat M.S., Harikumar K. (2018). Effects of Gyroscopic Coupling and Countertorque in a Fixed-Wing Nano Air Vehicle. J. Aircr..

[25] Blakelock J.H. (1991). Automatic Control of Aircraft and Missiles.

[26] Harikumar K., Joseph A., Bhat M.S., Omkar S.N. (2014). Static output feedback control for an integrated guidance and control of a micro air vehicle. J. Unmanned Syst. Technol..

[27] Oliveira M.C., Bernussou J., Geromel J.C. (1999). A new discrete-time robust stability condition. Syst. Control Lett..

